# Penetration of avian and human influenza A viruses through mucus gel networks in vitro

**DOI:** 10.1038/s44298-026-00257-7

**Published:** 2026-11-11

**Authors:** Maximilian Rohde, Fiona Riegel, Ege Duran, Cosmin Stefan Butnarasu, Daniel Christian Lauster, Christian Sieben

**Affiliations:** 1 Nanoscale Infection Biology Group, Helmholtz Centre for Infection Research, Braunschweig, Germany; 2 Institute of Pharmacy, Biopharmaceuticals, Freie Universität Berlin, Berlin, Germany; 3 Institut für Genetik, Technische Universität, Braunschweig, Germany; 4 BRICS - Braunschweig Integrated Centre of Systems Biology, Technische Universität, Braunschweig, Germany; 5 Present Address: Department of Molecular Biotechnology and Health Sciences, University of Turin, Turin, Italy

**Keywords:** Diseases, Microbiology

## Abstract

Influenza A viruses (IAVs) remain a public-health concern because of their continuous evolution and zoonotic transmission. Respiratory mucus is the first extracellular barrier encountered by inhaled viruses, yet standardized approaches for studying influenza virus passage through mucus and the infectivity of transferred virus populations remain limited. Here, we established an in vitro virus–mucus penetration assay to analyze IAV interactions with standardized mucin-containing matrices. We used mucus from human Calu-3 airway epithelial cells cultured under air–liquid interface conditions and bovine submaxillary mucin as surrogate matrices. Preparations were normalized by total protein and characterized using biochemical and biophysical methods. We compared a human seasonal H1N1pdm virus with a highly-pathogenic avian H5N1 virus. Viral genome transfer was quantified by RT-qPCR, while retained infectivity was assessed in susceptible cells. Both mucin-containing matrices restricted viral passage compared with control solutions. Viral RNA transfer and retained infectivity varied by matrix and virus, although no statistically significant difference between H1N1pdm and H5N1 was detected within either matrix. Together, these results establish a framework integrating mucus characterization with complementary measurements of viral transfer and infectivity. This approach provides a basis for investigating influenza virus–mucus interactions under controlled conditions and can be extended to other IAV strains and physiologically relevant mucus sources.

## Introduction

Influenza A viruses (IAVs) circulate worldwide and pose a persistent health threat to both humans and animals. Wild aquatic birds, particularly ducks and geese, constitute the principal natural reservoir of IAVs, while seasonal epidemics occur annually in the human population. These infections cause substantial morbidity and mortality, especially among vulnerable and high-risk groups, and impose considerable social and economic burdens. Vaccination remains the most effective measure for preventing severe influenza. However, the continuous evolution of IAVs can reduce vaccine effectiveness and necessitate regular updates of vaccine strains^1–3^. The emergence of influenza viruses with pandemic potential remains a persistent global threat. The ongoing spread of H5N1 clade 2.3.4.4b among migratory birds and diverse mammalian species, together with its recent emergence in dairy cattle in the United States, provides a striking example of this dynamic^4–9^.

In order for a new host to become infected via the respiratory route, viral particles must be inhaled or applied to the respiratory tract. An intrinsic defensive mechanism employed by the respiratory epithelia to prevent infection is the respiratory mucus layer. The subsequent mucociliary clearance is the primary defense mechanism that protects airways from inhaled infectious and also other toxic agents. The overall structure of the mucus layer that covers the cilia can be described as a “gel-on-brush” model. This model consists of a periciliary layer that optimizes ciliary beating and an overlaying mucus layer that protects cells from dehydration while ensuring good airflow^10,11^. Respiratory mucus constitutes a densely cross-linked hydrogel composed predominantly of MUC5AC and MUC5B mucins. It has been described that the mucins in particular facilitate the formation of the hydrogel, and that they possess proline-threonine-serine (PTS) domains with exposed hydroxyl groups where O-glycosylation can occur^12,13^. IAV binds to sialic acid containing receptors on host surfaces via its viral glycoprotein hemagglutinin (HA) protein, enabling initial attachment^14^. In mucus, these interactions occur within a dense and heterogeneous glycan network. Sialic acids, which are found within the mucus, act as decoy receptors for HA interaction and subsequent neuraminidase (NA) cleavage^15–19^. IAV strains from either avian or human origin exhibit distinct variations in HA receptor affinity and specificity. Most human-adapted IAV strains possess a binding preference for sialic acid with an α2,6 linkage to the underlying galactose. In contrast, avian IAV strains generally exhibit a binding preference for sialic acids with an α2,3 linkage^20,21^. Furthermore, the physical characteristics of the hydrogel, such as viscosity and pore size, influence the interaction between penetrating viral particles and mucus^22,23^. Virus-mucus interactions are complex and shaped by multiple biochemical and biophysical factors. The ability of zoonotic viruses such as IAV to cross species barriers may therefore also depend on the composition and structural diversity of mucins, which vary not only between host species but also among individuals within the same species^24–26^. Native respiratory mucus is a heterogeneous biological material whose composition and biophysical properties can vary according to anatomical location, physiological state, and individual donor. However, the collection of respiratory mucus from humans or cattle is technically challenging and may alter mucus hydration, composition, and rheological properties. Therefore, defined in vitro mucus models are frequently used to enable reproducible and mechanistically controlled comparisons. In the present study, we used two complementary mucin-containing model matrices rather than native respiratory mucus samples. Human mucus was obtained from Calu-3 cells differentiated under air-liquid interface conditions. This model produces secreted gel-forming mucins, including MUC5AC and MUC5B, and provides a standardized human airway epithelial source^27^. As an animal mucin model, we used commercially available bovine submaxillary mucin (BSM). BSM originates from the bovine submaxillary gland and should therefore not be considered equivalent to native bovine respiratory mucus^28^. Accordingly, the two preparations were used here as standardized surrogate matrices to investigate how distinct mucin-associated biochemical and biophysical environments influence IAV penetration and infectivity.

The composition and organization of respiratory mucus vary between host species and across anatomical regions. In the human respiratory tract, the gel-forming mucins MUC5AC and MUC5B are the primary structural mucins, produced by distinct secretory epithelial compartments^11,12^. Similarly, bovine respiratory secretions have been shown to contain large gel-forming mucins originating from the surface epithelium and submucosal tissue, alongside smaller soluble mucin populations^29^. Although several bovine mucin genes have been identified^30^, including bovine homologues of MUC5AC, MUC5B and MUC19, the tissue-specific expression, glycosylation and biophysical organization of bovine respiratory mucins are less well characterized than those of human airway mucins.

In the context of pandemic preparedness, it is important to understand how mucus acts as a host barrier and how passage through mucus affects viral infectivity. A better understanding of virus-mucus interactions may reveal determinants of host adaptation and transmission and could also inform the development of antiviral strategies and other anti-infective interventions. The objective of this study was to compare the mucus penetration capacity of the human-origin IAV A/California/4/2009 (H1N1) (H1N1pdm) with that of the highly pathogenic avian IAV A/black-headed gull/Ger-BW/2023 (H5N1), a representative of the panzootic clade 2.3.4.4b. To mimic the respiratory mucus barrier in vitro, we used two mucin-containing hydrogels derived from different mammalian species. Overall, our study established new experimental assays and investigated how standardized mucus model matrices affect the penetration and infectivity of human- and avian-origin IAVs.

## Results

**Fig. 1:**
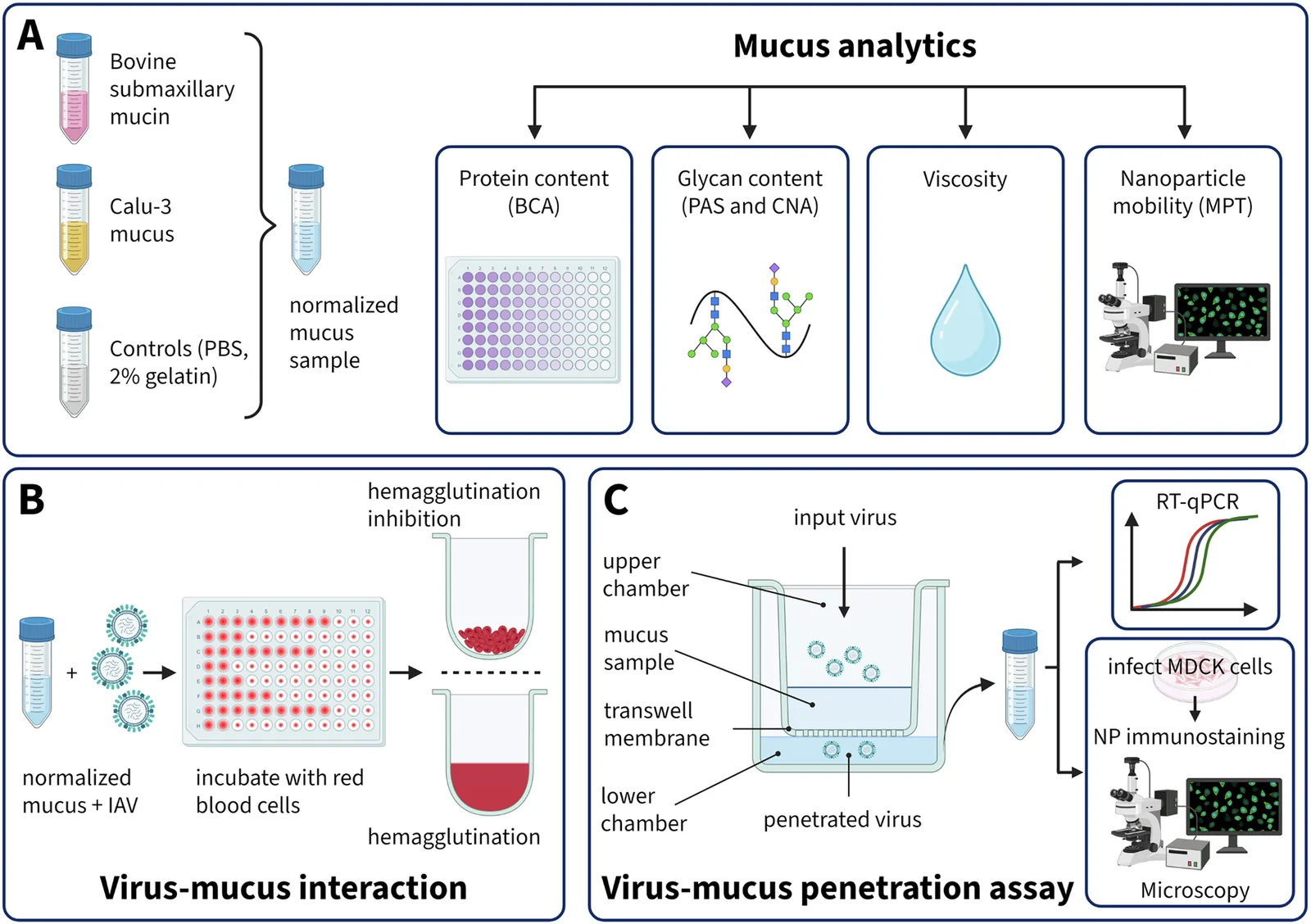
Experimental overview of our study on mucus and virus interactions. **A** Biochemical and biophysical characterization of human Calu-3-derived mucus, bovine submaxillary mucin, and control material, including protein and glycan content, bulk viscosity, and microscale particle mobility. **B** Hemagglutination inhibition assay using normalized mucus preparations as binding competitors for influenza A virus. **C** Virus–mucus penetration assay using a transwell insert. Mucus and virus were applied to the apical compartment, while the basolateral compartment contained culture medium. At the indicated time points, samples were collected from the basolateral compartment and analyzed in parallel by RT-qPCR for viral RNA and by infection of fresh MDCK cells. Infected cells were subsequently fixed, immunostained for influenza A virus nucleoprotein, and analyzed by fluorescence microscopy. Created in BioRender. Sieben, C. (2026) https://BioRender.com/w4p8ov2.

The central objective of our research was to address the knowledge gap surrounding virus-host interactions that occur before cellular infection. In the present study, we used two respiratory mucus model matrices: bovine mucus prepared from commercially available bovine submaxillary mucin (BSM) and human mucus collected from Calu-3 cell cultures. Both models contain secreted, gel-forming mucins, with the bovine model primarily representing MUC19 and the human model representing MUC5AC and MUC5B^27,28^. A 2% gelatin solution was included as a mucin-free viscosity control, while phosphate-buffered saline (PBS) served as a positive control for unrestricted viral passage. An overview of the different experimental assays performed in this study is shown in Fig. 1. In order to assess various mucus properties for normalization, we constructed an array of experimental assays, which were used to characterize the biochemical and biophysical properties of mucus. Subsequently, we looked at the interaction between viruses and mucus. Viral mucus penetration capacity was evaluated through the implementation of an assay configuration, in which a predetermined mucus layer was incorporated into an ALI culture insert. Subsequently, an IAV sample of a specified, pre-determined infection titer was introduced to the top of the insert. In the lower compartment, beneath the insert, the viral titers were then measured by RT-qPCR to assess the viral penetration. In parallel, viruses from the lower compartment were used to infect MDCK cells and evaluate the infectivity of the penetrated virus population. The configuration delineated herein was designated as the main model system, which we refer to as the virus-mucus penetration assay (VMPA, Fig. 1). In an additional control condition, the virus was introduced directly into the lower compartment (PosIN) to assess infectivity independently of mucus penetration. Here, the idea was to provide a value for the potential 100% penetration and virus stability over time.

### Mucus analytics and normalization

Initially, the protein and glycan content of the mucus samples was measured as outlined in the methods (Supplementary Fig. 1). To characterize their biophysical barrier properties further, we standardized the total protein concentration of Calu-3-derived human mucus and BSM-derived bovine mucus to 800 µg/mL. Then, we measured their apparent viscosity at shear rates of 3000, 6000, and 10,000 s⁻¹ at 37 °C using an m-VROC II microfluidic viscometer. We included a 2% (w/v) gelatin solution as a control. Under all tested conditions, the bovine mucus preparation exhibited lower apparent viscosity than the human mucus matrix. Meanwhile, the gelatin control exhibited values closer to those of the human mucus matrix (Table 1). An increase in shear rate resulted in a moderate reduction in the apparent viscosity of the mucus samples, which is consistent with the shear-dependent behavior described for BSM and other mucin-containing mixtures^28,31–33^. However, this effect was not evident for the gelatin control. The lower viscosity of the bovine-derived mucus may reflect differences in mucin composition and molecular organization and the presence of additional secreted components in the Calu-3-derived human preparation. However, the present measurements do not allow these mechanisms to be distinguished.

**Table 1:** Viscosity measurements of mucus samples and control material

Mucus Type	Method	Protein Content (µg/mL)	Shear Rate (1/s)	Temperature (°C)	Mean Viscosity (mPa-s)	SD
Bovine Mucus	m-VROC II	800	3000	37	0.988	1.28E–02
Human Mucus	m-VROC II	800	3000	37	1.496	1.59E–02
Gelatin 2%	m-VROC II	800	3000	37	1.475	1.32E–02
Bovine Mucus	m-VROC II	800	6000	37	0.966	1.23E–02
Human Mucus	m-VROC II	800	6000	37	1.405	3.54E–03
Gelatin 2%	m-VROC II	800	6000	37	1.481	8.01E–03
Bovine Mucus	m-VROC II	800	10,000	37	0.937	9.09E–03
Human Mucus	m-VROC II	800	10,000	37	1.337	1.15E–02
Gelatin 2%	m-VROC II	800	10,000	37	1.474	1.90E–03
Bovine Mucus	MPT beads	800		37	5.956	1.79E + 01
Human Mucus	MPT beads	800		37	4.108	5.11E + 00

Viscosity of BSM-derived bovine mucus, Calu-3-derived human mucus, and gelatin was determined using a microfluidic viscometer (m-VROC II, RheoSense). In parallel, viscosity was independently assessed by multiple particle tracking (MPT) analysis followed by calculation of dynamic viscosity values. Data are reported as mean values derived from *n* = 6 technical replicates from one preparation that was used for all experiments for the microfluidic measured viscosity. Shear rates were predefined instrument settings. The data therefore describe technical variability within the tested preparations and should not be interpreted as representing biological variability. Viscosity is reported in millipascal-seconds (mPa-s). In the case of the MPT dataset, the measurement was executed on multiple videos and trajectories on two separate occasions.

### Virus-mucus penetration assay

**Fig. 2:**
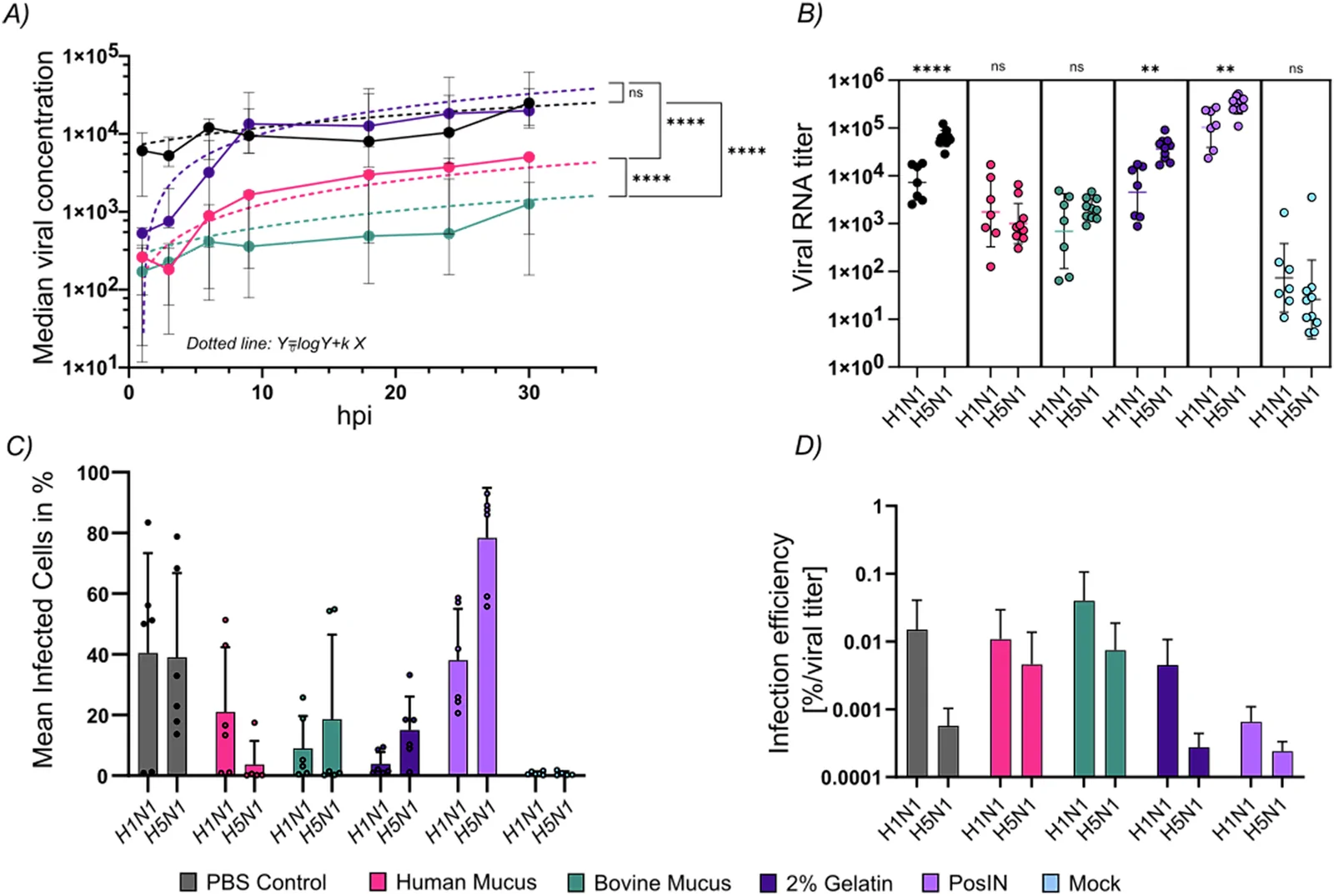
Virus penetration through mucus and downstream infectivity. **A** Time-dependent penetration of H1N1pdm influenza A virus through PBS, 2% gelatin, to 800 µg/mL protein standardized bovine and human mucus, shown as the median viral RNA titer concentration at the indicated time points (1, 3, 6, 9, 18, 24, 30 h). The initial viral titer was 5 × 10⁵ FFU in 20 µL added to the apical compartment. Data are shown as median with error and 95% CI from *n* = 3 independent experiments. Statistical significance was assessed using Kruskal-Wallis test on the non-linear fit. **B** Viral RNA detected in the basolateral compartment 24 h after addition of H1N1pdm or H5N1 to the respective matrix, quantified by RT-qPCR and expressed as FFU equivalents based on a virus-stock-derived standard curve. Viral RNA levels represent genome transfer through the matrix and do not directly indicate infectivity. Data are shown as mean with SD from three independent experiments. **C** Infectivity of the virus population recovered from the basolateral compartment after 24 h of passage through the respective matrix. Samples were transferred to MDCK cells, and infection was quantified by NP immunofluorescence and automated image analysis using a CellProfiler pipeline. Data are shown as mean with SD from three independent experiments. **D** Normalized infection efficiency, calculated as the fraction of NP-positive MDCK cells normalized to the corresponding viral titer qPCR. Data in (**B–D**) are shown as mean with SD from *n* = 3 independent biological experiments. Statistical significance between virus strains in (**B**) was assessed using an unpaired t-test. The *p*-values indicated by asterisks (*p < 0.05, **p < 0.01, ***p < 0.001, ****p < 0.0001).

Viral penetration through mucus was assessed using a transwell-based virus-mucus penetration assay (VMPA). We quantified the time-dependent penetration of H1N1pdm IAV particles through the different samples using VMPA. All VMPAs were performed with mucus samples diluted to 800 µg/mL protein concentration. We measured the median viral RNA titer over a 30 h time course in the lower compartment of the VMPA (Fig. 2A). Across three independent experiments, we observed that the penetration speed of H1N1pdm IAV was different in mucus types with the same standardized protein amount. H1N1pdm penetration was more effectively hindered by bovine-derived mucus compared to human mucus. Compared to the 2% gelatin and PBS control, both mucus samples interfered more strongly with viral penetration over time. To compare the penetration of the two IAV strains, we quantified viral RNA in the basolateral compartment 24 h after virus application. Both mucus matrices resulted in lower viral RNA transfer than the PBS and gelatin controls for H1N1pdm and H5N1 (Fig. 2B). Within the control conditions, H5N1 showed higher viral RNA levels than H1N1pdm, indicating more efficient genome transfer under these conditions. However, this difference was not statistically significant in either the Calu-3-derived human mucus or BSM-based mucus matrix. Thus, the data do not support a statistically significant strain-specific difference in viral genome penetration through the mucus matrices under the conditions tested. In the two mucus models, a clearly reduced transfer was observed for both virus strains in comparison to the control groups.

Mean viral infectivity following mucus penetration was assessed in parallel by infecting MDCK cells with virus-containing medium after 24 h passage through the VMPA setup. Infection was quantified by immunostaining against the IAV nucleoprotein (NP) 6 h post-infection (hpi) and subsequent microscopic analysis. The percentage of NP-positive cells was determined using an automated image analysis pipeline implemented in CellProfiler 4^34^. Penetration through Calu-3-derived human mucus resulted in a stronger reduction of infectivity for avian-origin IAV H5N1 compared to H1N1pdm IAV. In contrast, penetration through BSM-derived mucus preferentially reduced infectivity of H1N1pdm IAV relative to H5N1 IAV (Fig. 2C). Therefore, viral passage through the Calu-3-derived human mucus and BSM-based mucus matrices resulted in a reduction of NP-positive cells compared with the PBS control, with matrix-dependent differences in the magnitude of this effect. The mean NP-positive cell fraction differed between H1N1pdm and H5N1, but these differences were not significant in the respective mucus matrix. The 2% gelatin viscosity control reduced the infectivity of both H1N1pdm and H5N1 compared with the PBS control, with a more pronounced effect on H1N1pdm. This control was included to assess the influence of particle size, viscosity, and a non-binding physical barrier on viral passage and infectivity. Further analysis using the PosIN control revealed marked differences between H1N1pdm and H5N1, with H1N1pdm exhibiting substantially lower infectivity than H5N1. Please note, the low proportion of virus transferred through the relatively large mucus volume resulted in low infectious-virus recovery and large experimental variability, limiting the statistical power of the infectivity analysis. Finally, we normalized the infectivity (Fig. 2C) to the fraction of penetrated viral RNA (Fig. 2B) resulting in the overall infection efficiency, reflecting the fraction of viral genomes that resulted in productive infection of MDCK cells (Fig. 2D). In comparison with H1N1pdm, H5N1 demonstrated a reduced infection efficiency across all experimental conditions, despite equivalent initial viral titers (5 × 10⁵ FFU in 20 µL). In the case of H1N1pdm, the penetration of most tested barriers resulted in a reduction in infection efficiency relative to the PBS control. The only exception was BSM-derived bovine mucus, where a slight increase in infection efficiency was observed. In contrast, H5N1 showed reduced tendency in infection efficiency in the PBS, gelatin, and PosIN control conditions, whereas infection efficiency increased in both mucin-containing conditions. These findings suggest that the presence of mucins differentially modulates the infectivity of H1N1pdm and H5N1 following mucus barrier penetration. The analysis indicated matrix-dependent differences between H1N1pdm and H5N1. These findings should be interpreted as exploratory and not as evidence of a strain-specific effect.

### Virus-mucus interaction and neuraminidase activity

**Fig. 3:**
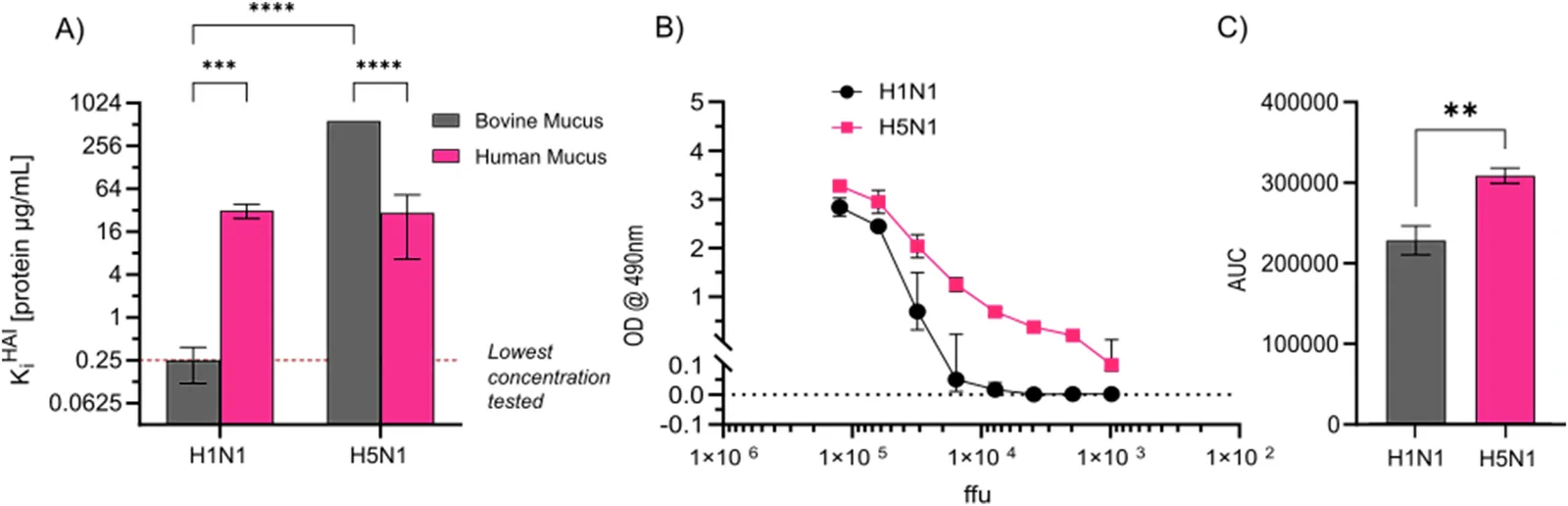
Mechanistic analysis of HA inhibition and neuraminidase activity. **A** Inhibition of hemagglutination was quantified using O-glycan-normalized samples and expressed as the inhibitory constant K_i_HAI for H1N1 and H5N1 influenza A virus under the indicated conditions. This readout reflects the net outcome of virus–mucus–erythrocyte interactions and is influenced by HA-mediated receptor binding, NA-mediated receptor cleavage, and glycan presentation. **B** Neuraminidase activity was assessed by enzyme-linked lectin assay (ELLA), and NA activity is shown as OD measured at 490 nm. The assay provides a relative measure of virus-associated NA activity under the tested conditions. **C** Baseline NA activity of H1N1 and H5N1 influenza A virus was measured in the absence of inhibitors and quantified as area under the curve (AUC) to summarize the data from panel **B** in a single value. Data are shown as mean with SD from *n* = 3 independent experiments. Statistical significance was assessed (**A**) using two-way ANOVA with Tukey’s multiple-comparisons test or (**C**) Welch’s t-test, with significance indicated as *p < 0.05, **p < 0.01, ***p < 0.001, ****p < 0.0001.

To identify potential reasons for the observed VMPA results, we next investigated the interaction between mucus and the two IAV strains as well as their enzymatic neuraminidase (NA) activity. To assess the net effect of virus–mucus interactions on virus-mediated hemagglutination, we titrated the mucus preparations in a hemagglutination inhibition assay (see methods). The resulting ${K}_{i}^{{HAI}}$ values reflect the concentration of mucus required to inhibit hemagglutination under the assay conditions. This readout is influenced by the combined effects of viral receptor binding, neuraminidase-mediated receptor cleavage, and the glycan composition of the mucus and erythrocyte surfaces. Low ${K}_{i}^{{HAI}}$ values indicate strong HA inhibition, whereas high ${K}_{i}^{{HAI}}$ values reflect weak inhibitory activity (Fig. 3A). The added mucin concentrations for the assay were standardized to the same O-glycan (3 × 10⁴ RFU) content and subsequently calculated to their respective protein content at this concentration. This approach was taken to harmonize the binding efficiency with respect to the available mucin glycans, which we hypothesize to represent competitive binding sites for IAVs.

For both virus strains, significant differences were observed between bovine and human-derived mucus treatment. Both virus strains showed similar ${K}_{i}^{{HAI}}$ values after treatment with human mucus. However, the hemagglutination inhibition between the IAV strains differed strongly for bovine-derived mucus. For H1N1pdm, even low concentrations of BSM-derived bovine mucus were found to be effective in inhibiting viral hemagglutination. For avian-origin H5N1 IAV, a significantly higher concentration of mucus was required for hemagglutination inhibition in comparison to H1N1. These results indicate that the two virus strains showed comparable net sensitivity to inhibition by the Calu-3-derived mucus preparation under the assay conditions, while H1N1pdm is significantly more sensitive to BSM-derived bovine mucus compared to H5N1 (see also Fig. 2A). These results suggest that H1N1pdm is more susceptible than H5N1 to hemagglutination inhibition by BSM-derived bovine mucus.

The IAV NA reduces the amount of sialylated glycans available for HA to bind and thus its activity could have an effect on the observed differences in hemagglutination. We thus quantified the activity of NA using a colorimetric enzyme-linked lectin assay (ELLA) with serial viral dilutions (Fig. 3B). The concentration-dependent optical density (OD) was measured and displayed as a curve, and the area under the curve was subsequently measured. This analysis showed significant differences between the two viral strains (Fig. 3C). The avian-origin H5N1 strain showed higher relative virus-associated sialidase activity in the ELLA than H1N1pdm at the tested virus input. However, despite the observed elevated NA activity, the overall H5N1 penetration and permeability did not exceed those of the H1N1pdm (Fig. 2 B). The ELLA provided a relative measure of the sialidase activity of intact virus particles under the assay conditions. Although this assay is primarily used to quantify viral NA activity, the measured signal may also be influenced by substrate accessibility, virion abundance, and the functional organization of HA and NA on the viral surface.

### Multiple particle tracking

**Fig. 4:**
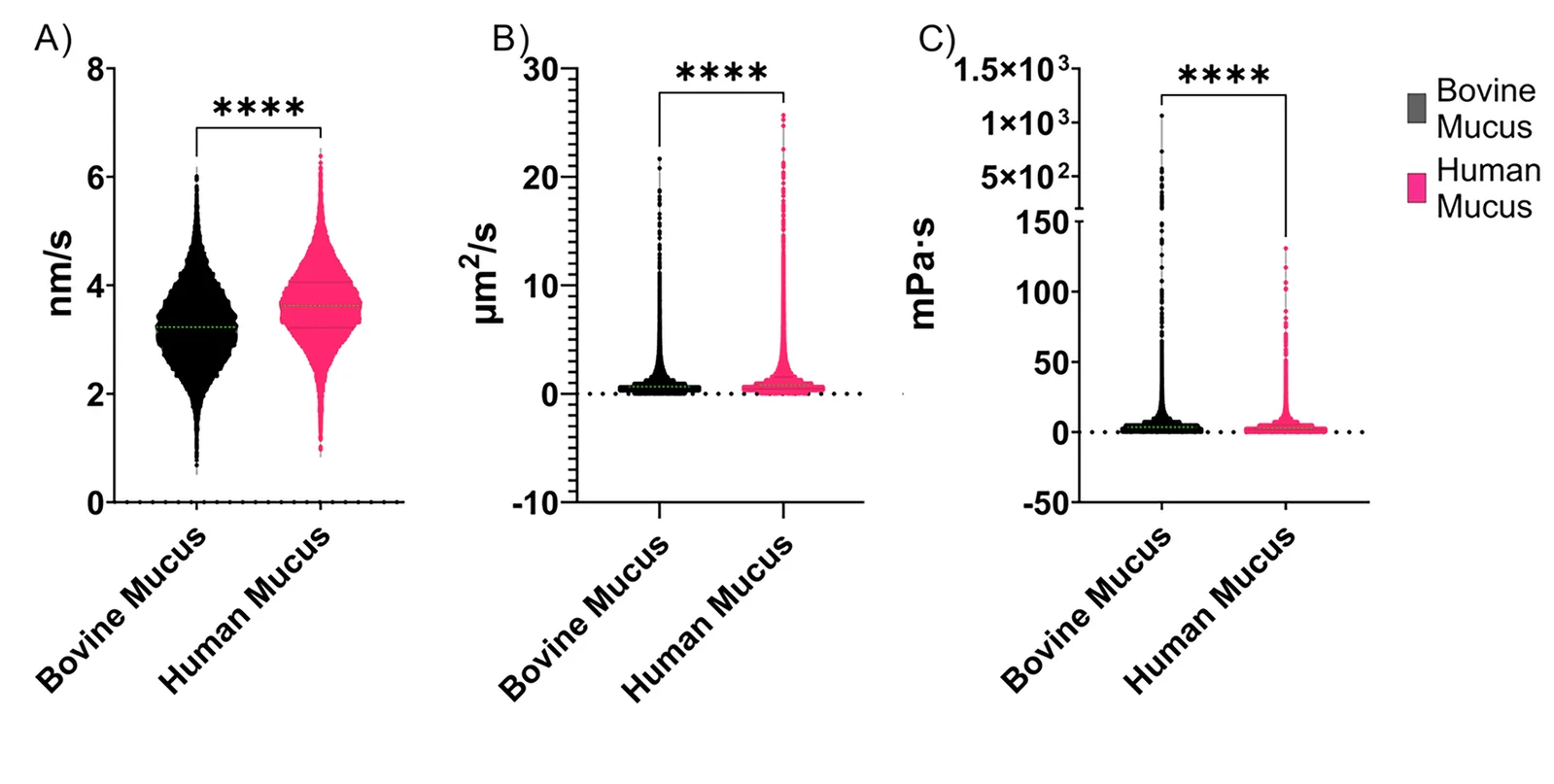
Particle mobility and viscosity measurements in mucus by multiple particle tracking. Multiple particle tracking (MPT) was performed using fluorescent beads in human (Calu-3–derived) mucus or bovine submaxillary mucus (BSM) at protein concentrations of 800 µg/mL. Graphs **(A**–**C)** are shown as violin plots with individual measurements indicated as dots. Statistical significance was assessed using the Mann–Whitney U test, with *p*-values indicated by asterisks (*p < 0.05, **p < 0.01, ***p < 0.001, ****p < 0.0001).

We reasoned that IAV penetration through mucus is primarily governed by two factors: mucin binding and the mechanical properties of the mucus meshwork. To isolate the latter, we used nanoparticles that do not bind to mucus specifically and can therefore report on differences in meshwork properties between samples. We performed multiple particle tracking (MPT) with fluorescent nanoparticles with a diameter of 200 nm. In contrast to IAV, the nanoparticles primarily reflect passive particle diffusion within mucus and lack specific glycan-binding or enzymatic interactions with mucins. Particle tracking was performed in mucus at 800 µg/mL protein content using widefield fluorescence microscopy. Mean squared displacement (MSD) analysis revealed a linear dependency across short distances indicating diffusive particle movement in both mucus models. The mean nanoparticle speed in BSM-derived bovine mucus was lower (median 3.15 nm/s) than the speed observed in human mucus (median 3.626 nm/s) (Fig. 4A). Similarly, the bead diffusivity in bovine mucus was also lower (median 0.652 µm^2^/s) compared to human mucus (median 0.813 µm^2^/s) (Fig. 4B). From the diffusivity, we calculated the dynamic viscosity (η) using the Stokes-Einstein-Sutherland relation. The calculation is primarily dependent on the previously calculated particle diffusivity. We found a significantly higher median dynamic viscosity of bovine mucus compared to human mucus (Fig. 4C and Tab.1). Overall, these data indicate that meshwork properties lead to a more pronounced slow-down of nanoparticles in bovine mucus.

## Discussion

In this study, we established an in vitro virus-mucus penetration assay to compare the mucus passage of different IAV strains including the human-derived H1N1pdm and the avian-origin H5N1 IAVs through mucin-containing gel matrices.

Our VMPA assay permitted the measurement of IAV mucus penetration over time and in parallel we assessed the effect of mucus transfer on the viral infectivity. Overall, we found that viral penetration through mucus was markedly slower compared to PBS or 2% gelatin (Fig. 2). Similar findings have been reported previously and can be explained by the interplay of the viral HA and NA with mucin-containing mucus^35^. Mucus, with its O-glycan-rich mucins, has been described to capture pathogens and facilitate subsequent mucociliary clearance from the respiratory tract^36^. The balance of the HA/NA-mucus interaction is therefore crucial for viral fitness, as it prevents viral immobilization and subsequent mucociliary clearance^18,37^. In other instances, mucus, in its role as a dense hydrogel, has been shown to interfere with particle penetration, a phenomenon that is the subject of ongoing scientific discourse^22,38,39^. Our VMPA allows for the assessment of the combined influences of physical IAV restriction by the mucus network as well as the biochemical interactions of their respective HA with the mucins and subsequent cleavage through the viral NA.

Figure 2C shows the viral infectivity of H1N1pdm and H5N1 after mucus penetration. The infectivity of both tested IAV strains was reduced compared to the PBS control, exhibiting divergent trends towards the mucus sources. Indeed, previous studies have indicated that mucus can reduce viral infectivity and our data are consistent with this observation^35,40^. We observed a matrix-dependent trend in the tested model systems in the effects of mucus on viral infectivity: H5N1 was more strongly impaired by the human mucus model, whereas H1N1pdm was more strongly affected by the bovine mucus model. Passage through the gelatin control also reduced infectivity, despite the absence of specific virus-mucin interactions. This effect was more pronounced for H1N1pdm than for H5N1 and was consistent with the trend observed for the bovine mucus model (Fig. 2C).

To determine whether the observed reduction in IAV infectivity was solely attributable to impaired transport through the mucus layer, we quantified viral RNA levels after mucus penetration (Fig. 2B). The infectivity of IAV appears to be associated with the viral RNA titers, with higher values resulting in increased infectivity. It is noteworthy that even in instances of elevated viral RNA titers within the gelatin control, the level of infectivity was reduced, falling slightly below the levels observed in mucin-containing setups. This suggests that viral particles may penetrate the layer more rapidly, but they may also be subject to degradation. In order to achieve a more in-depth understanding of the results, the data were normalized to facilitate the visualization of overall infection efficiency (transfer and infection efficiency) (Fig. 2D). As shown by the PBS control, a substantial fraction of viral particles crossed the membrane and remained capable of infecting MDCK cells. Interestingly, the normalized infection efficiency was particularly low for H5N1 in the PBS condition but increased in both mucus models. This suggests that mucus may not only protect the underlying epithelial cells by restricting viral access, but may also partially preserve viral stability and infectivity during extracellular exposure^41,42^.

A different indicator of viral fitness in an in vivo setting is the speed of penetration to avoid mucociliary clearance. A general observation suggests that the rate of H5N1 viral particle transfer is higher in experimental controls (Fig. 2B). Nevertheless, within mucus-containing setups, these differences are less pronounced and are only visible as trends. Recent studies suggested that the mucus mesh structure restricts the diffusion of the IAV more than glycan-binding interactions^22,23^. Our data do not distinguish whether mucus mesh size or virus-mucin binding is the dominant factor. However, strain-specific binding differences were detectable, and direct particle tracking of virions in mucus could clarify their contribution to mobility and confinement. H5N1 and H1N1pdm also showed different infection efficiencies after mucus penetration. In our static model, significant differences were mainly observed at early time points, but these may become more relevant under dynamic mucociliary transport, where even short delays could enhance viral clearance. The observed differences are best described as matrix-dependent trends rather than definitive strain-specific or host-specific effects. In particular, the present data do not establish that H5N1 penetrates human or bovine mucus more efficiently than H1N1pdm, nor that one virus is generally more strongly restricted by one mucus source.

IAV surface proteins HA and NA interact with mucus, which determines the effectiveness of viral infections^15,18,35,43^. To better interpret our previous findings, we therefore examined the inhibitory effects of the standardized mucus preparations on virus-mediated erythrocyte hemagglutination. As shown in Fig. 2B, higher viral RNA levels were detected for H5N1 than for H1N1pdm. This is consistent with the finding that the tested mucin concentrations exerted a weaker inhibitory effect on H5N1, as indicated by the hemagglutination assay in Fig. 3A. In contrast, H1N1pdm was particularly sensitive to BSM-derived bovine mucus, in agreement with its low penetration efficiency in the VMPA (Fig. 2A). Notably, mucus derived from Calu-3 cells affected all tested IAV strains to a similar extent. Overall, these results indicate that the impact of mucus is strongly dependent on both the viral strain and the origin of the mucus^19,44^.

Taken together, the VMPA data (Fig. 2), hemagglutination inhibition results (Fig. 3A), and viscosity measurements (Table 1) indicate that HA-mucus interactions contribute substantially to viral penetration. In particular, H1N1pdm was more strongly affected by BSM-derived bovine mucus than by Calu-3-derived human mucus. The two principal strains investigated in this study, H5N1 and H1N1pdm, also showed markedly different sensitivities to hemagglutination inhibition. These differences may reflect variation in the mucin composition and glycosylation patterns of the two mucus models, which could alter their barrier properties. An integrated analysis of a dynamic infection model including mucus composition, biophysical properties, viral binding, and penetration is therefore required to determine how the respiratory mucus barrier contributes to IAV host range and cross-species transmission.

With respect to neuraminidase activity, H5N1 showed significantly higher overall activity than H1N1pdm. However, the VMPA data indicate that this elevated activity did not translate into a corresponding increase in H5N1 penetration or permeability compared with H1N1pdm under our experimental conditions (Fig. 3B). The assays used in Fig. 3 should be interpreted as functional assays of intact virus particles rather than as isolated measurements of HA and NA activity. The HAI assay quantifies the net inhibition of virus-mediated erythrocyte agglutination by the mucus preparations. Its outcome depends on HA-mediated receptor binding, NA-mediated cleavage of sialic acids, and the receptor composition of the mucus and erythrocyte surfaces. Likewise, the ELLA provides a relative measure of virus-associated sialidase activity using fetuin as a substrate, but the signal may also be influenced by virion abundance, substrate accessibility, and the functional organization of HA and NA. Previous studies have shown that neuraminidase activity can facilitate influenza virus movement through sialylated mucus by cleaving mucin-associated decoy receptors. Conversely, pharmacological inhibition of NA with zanamivir or oseltamivir can increase virus retention in mucus and reduce viral movement or infection^18,23,37^. To understand which IAV particles are able to traverse the mucus barrier, it is thus necessary to consider both virus-mucin interactions and the biophysical properties of mucus, including bulk and microscale viscosity, steric hindrance, and viscoelastic behavior. Because mucus rheology varies with shear rate, shear stress, and temperature^33^, we characterized these properties to assess the quality and comparability of our mucus models (Table 1). The viscosity measurements should be interpreted in the context of the composition and preparation of the respective model matrices. Although the samples were normalized to the same total protein concentration, this normalization does not establish equivalence in mucin concentration, mucin molecular weight, glycosylation, hydration, or network organization^28,33^. The Calu-3-derived human mucus preparation represents an epithelial secretion containing gel-forming mucins and potentially additional secreted non-mucin components. In contrast, BSM-derived bovine mucus preparation is a commercially available and processed submaxillary mucin preparation^12,28^. The lower apparent viscosity may therefore result from differences in mucin composition and molecular organization, as well as from the effects of purification, dilution, and reconstitution. The present measurements do not allow these mechanisms to be distinguished. The viscosity difference is relevant for characterizing the standardized matrices used in the VMPA, but it should not be interpreted as a direct quantitative comparison of native bovine and human respiratory mucus. Moreover, bulk viscosity represents only one aspect of mucus barrier function. Particle mobility and viral transport may also be affected by microscale mesh structure, steric interactions, mucin binding, glycan presentation, and viral HA–NA activity^22,23,40^. Consistent with this interpretation, the bulk viscosity measurements did not fully predict the particle-tracking or virus-penetration behavior of the tested matrices (Fig. 4). These findings indicate that bulk viscosity alone does not capture the microscale transport properties that determine viral mobility through mucus. Further research could entail an examination of fluorescent beads that have been coated with either IAV HA or NA with the objective of discerning their role in the process of mucus penetration. The filamentous IAV morphology has recently been linked to enhanced aerosol stability and reduced mucus-mediated restriction, suggesting that virion shape may contribute to transmission fitness^39^. In the future, the use of a cattle-associated H5N1 virus, such as a B3.13 genotype virus, would be highly interesting for investigating bovine host adaptation. However, this would adress a distinct biological question. Cattle-associated H5N1 viruses have been associated with pronounced mammary gland infection and transmission linked to milk and milking procedures, whereas the present study focused on the interaction of an avian-origin virus with standardized mucus matrices^45^. Mucus differences between species may be due to differences in mucin types, amounts, glycosylation, molecular size, and gland contributions. Human airway mucus is well-characterized, especially regarding MUC5AC and MUC5B. In contrast, the composition and organization of bovine respiratory mucus is less well understood. While bovine tracheal secretions contain gel-forming mucins from both epithelial and submucosal glands, these native secretions are not equivalent to the BSM used in this study^28,29^.

Together, our findings show that virus-mucus interactions are multifaceted and can influence both viral transport and infectivity. However, the experimental systems used here remain reductionist models of the native respiratory mucus environment and cannot fully reproduce its structural and biochemical complexity. The Calu-3-derived preparation provided a controlled, reproducible model of human airway epithelial mucus, while the BSM provided a standardized matrix containing bovine mucin. However, BSM is derived from the bovine submaxillary gland and therefore does not fully replicate the composition of bovine respiratory mucus. Similarly, Calu-3-derived mucus is produced by a transformed epithelial cell line and does not reproduce the full cellular, proteomic, lipid, immune, and structural complexity of native human airway mucus. Normalization to equal total protein concentration, along with the biochemical and biophysical characterization in this study, enabled controlled comparisons between the preparations. However, this normalization does not imply biological equivalence, since the relative abundance and molecular organization of mucins, glycans, non-mucin proteins, lipids, and other mucus components may remain different. Thus, the observed differences should be interpreted as properties of the respective standardized model matrices rather than as direct quantitative differences between native human and bovine respiratory mucus. Future studies should therefore examine a broader range of mucin concentrations and compositions and further investigate the apparent protective effect of mucus on H5N1 stability. Furthermore, the utilization of native mucus, for relevant species would serve as an exemplary model for conducting a more comprehensive investigation. Zoonotic viruses such as IAV must traverse the respiratory mucus layer before reaching susceptible epithelial cells. Because mucin composition and structure vary between host species, these differences may contribute to host restriction, viral adaptation, and cross-species transmission. Our findings are consistent with previous studies showing that respiratory mucus can restrict viral access to epithelial target cells while also modulating the stability and infectivity of virus particles during extracellular exposure. Rather than introducing this concept as a novel observation, our study provides a standardized in vitro framework in which viral passage through defined mucin-containing matrices can be assessed together with the infectivity of the transferred virus population. The observed matrix-dependent differences highlight the need for further studies using native mucus and primary airway models to determine how mucus composition, structure, and virus–mucin interactions influence influenza virus infection and transmission in vivo.

## Methods

### Cells and viruses

MDCK II (obtained from Thorsten Wolff, Robert-Koch Institute) and Calu-3 (obtained from Thorsten Wolff, Robert-Koch Institute) cells were maintained in Dulbecco’s modified Eagle medium (DMEM) supplemented with L-glutamine and 10% (v/v) fetal bovine serum (FBS). For Calu-3 cells, the culture medium was additionally supplemented with non-essential amino acids (NEAA). Cells were cultured at 37 °C in a humidified incubator with 5% CO₂ and routinely passaged using standard cell culture procedures. For air-liquid interface (ALI) differentiation, Calu-3 cells were seeded onto permeable transwell inserts (CellQART Ref. 9320412) with a pore size of 0.4 µm and a growth area of 0.3 cm² and grown under submerged conditions until confluence. Apical medium was subsequently removed to establish ALI conditions, and cells were differentiated for 21 days with regular medium exchange in the basolateral compartment. Influenza virus stocks were propagated in embryonated chicken eggs following standard procedures. Briefly, fertilized eggs were incubated under controlled conditions for 10 days and subsequently inoculated with virus via the allantoic cavity. After incubation for 48 h at 37 °C, allantoic fluid containing progeny virus was harvested, clarified by centrifugation, aliquoted, and stored at –80 °C until further use. For virus titration, susceptible cell monolayers were inoculated with serial virus dilutions. Following virus adsorption, the inoculum was removed and the cells were incubated under an overlay containing 2.5 μg/mL trypsin-NAT (Merck T6763) for an additional 24 h at 37 °C and 5% CO₂. The overlay was then carefully removed, and the cells were washed twice with phosphate-buffered saline (PBS), fixed with 4% paraformaldehyde, and immunostained for viral nucleoprotein. Infectious virus titers were calculated as focus-forming units (FFU) based on the number of NP-positive foci and the corresponding dilution factor. The following influenza A virus strains were used in this study: A/California/4/2009 (H1N1pdm, Source: Robert-Koch Institute, EPI176620), and A/black-headed gull/Ger-BW/2023 (H5N1, Source: Friedrich-Loeffler Institute, EPI18014047). Virus stocks were propagated and handled in accordance with institutional biosafety regulations and national guidelines. Experiments involving H1N1pdm were performed under biosafety level 2 (BSL-2) conditions in certified biosafety cabinets. Experiments involving H5N1 were conducted under biosafety level 3 (BSL-3) conditions in a high-containment laboratory approved for work with highly pathogenic influenza viruses. All procedures involving live virus were carried out by trained personnel following approved biosafety protocols.

### Cell-derived mucus isolation

Calu-3 cells were seeded on permeable transwell inserts and maintained under submerged conditions until confluence. The apical medium was then removed, and the cultures were maintained at an air-liquid interface for at least 21 days to permit differentiation and mucus accumulation. The apical surface was covered with 100 µL PBS prewarmed to 37 °C. After 15-20 min, the apical wash was collected carefully. Samples were clarified by centrifugation at 300 x g for 5 min at 4 °C, then aliquoted and stored at −80 °C while avoiding repeated freeze-thaw cycles. The resulting preparation was used as a standardized human airway epithelial mucus model rather than as a substitute for native human respiratory mucus.

### Lyophilized mucus sources

Bovine submaxillary mucin [28] (BSM; Merck; 499643-500MG) was utilized as a bovine mucus model. The substance was reconstituted in PBS at a concentration of 10 mg/mL, subjected to protein and glycan quantification, and used in our experiments under the same standardized conditions. In addition, a 10% (w/v) stock concentration of gelatin was prepared, and its viscosity at 37 °C was matched to that of the other types of mucus, which was approximately 2% (w/v). This was employed as a binding-free viscosity control in the experimental setup.

### Mucus analytics and normalization

For the assessment of mucus quality and following standardization, we performed multiple analytics. First, we analyzed the protein content with a commercial bicinchoninic acid assay (BCA, ThermoFisher), a Periodic acid–Schiff (PAS) assay to analyze the mucin concentration, followed by an O-glycan quantification assay for quantification of O-linked glycoproteins. Prior to analysis, mucus samples were clarified by centrifugation and diluted in phosphate-buffered saline (PBS) to ensure measurements within the linear range of the assays. Protein quantification: Total protein concentrations in mucus samples were determined using a BCA assay according to the manufacturer’s instructions. A standard curve was generated using bovine serum albumin (BSA) prepared in the same buffer matrix. Absorbance was measured at 562 nm, and protein concentrations were calculated from the standard curve. For all downstream analyses, mucus samples were normalized to an equal total protein concentration as determined by the BCA assay. Periodic acid–Schiff (PAS) assay**:** Total carbohydrate content in mucus samples was assessed using a periodic acid–Schiff (PAS) assay. Protein-normalized mucus samples were incubated with 0.06% (w/v) periodic acid in 7% acetic acid to oxidize vicinal diols, followed by reaction with Schiff reagent according to published protocols^46^. Absorbance was measured spectrophotometrically at 555 nm, and PAS signals were used as a relative measure of total glycoprotein content.

O-glycan quantification: The O-glycan content of mucus samples was quantified using a fluorescence-based alkaline cyanoacetamide (CNA) assay^47^. Samples were incubated under alkaline conditions in the presence of 2-cyanoacetamide. This released and derivatized the O-linked glycans, which were detected by fluorescence measurement. After neutralization, fluorescence was measured in 96-well plates using excitation and emission wavelengths respectively of 336 and 383 nm. Fluorescence signals were background-corrected and expressed as relative fluorescence units (RFU) of total O-glycan content. Each sample was analyzed in technical triplicates, and positive (fetuin) and negative (PBS) controls were included. O-glycan levels were expressed relative to total protein content and used for normalization in the subsequent functional assay, hemagglutination inhibition (HAI).

### Viscosity measurements

The viscosity measurements were performed with mucus and gelatin Type B (Merck, G9391). The concentrations of the BSM-derived bovine mucus matrix and the Calu-3 cell-derived human mucus matrix were normalized to 800 µg/mL total protein. The viscosity of gelatin

was assessed using a 2% (w/v) concentration which served as a control. Mucus and gelatin were diluted with phosphate-buffered saline (PBS). Measurements were performed at shear rates of 3000, 6000, and 10,000 s⁻¹ and at a temperature of 37 °C using an m-VROC II microfluidic viscometer (RheoSense). Data are reported as mean Viscosity (mPa-s) with standard deviation (SD) of the technical measurements. Because only one independent preparation or reconstruction was analyzed per matrix, these data were used for descriptive characterization of the tested model matrices and not for inference on biological variation.

### Multiple particle tracking

Mucin or gelatin solutions were generated using phosphate-buffered saline (PBS). Fluorescently labeled 200-nm PMMA beads (PolyAn #10910009) were added to the mixture, which was then thoroughly homogenized. For the beads, a concentration of 0.002% was used. Fluorescent 200-nm PMMA beads were selected as passive probes because this particle size is within the relevant length scale for particle transport through respiratory mucus and is comparable to the size range of influenza virions. The sample was equilibrated at 37 °C for 20 min. Subsequently, the sample was subjected to a second round of homogenization via pipetting, after which it was transferred to a silicon chamber on a microscopy slide. The samples were imaged using a high-resolution microscope equipped with an Apo TIRF 100x Oil DIC N2 objective, with a 100x magnification setting and at a temperature of 37 °C. The Andor DU-897 X-6449 camera was utilized with an exposure time of 20 milliseconds (ms). In order to analyze the movement of the particles, their location in each frame was determined, and subsequently, they were linked into trajectories. In this particular case, the locating process was executed with the aid of a TrackMate algorithm that leveraged the Laplacian of Gaussian (LoG) detector, operating under the assumption that the object’s diameter was estimated to be 1 µm. The particle’s locations were linked using Python’s Trackpy. Subsequently, the ensemble mean squared displacement (eMSD) was calculated. The eMSD is employed to retrieve information regarding the medium’s dynamic viscosity and the particle’s diffusivity.

### Viral mucus penetration assay (VMPA)

Viral penetration through mucus was assessed using a transwell-based virus-mucus penetration assay. Mucin samples were thawed immediately prior to use, clarified by centrifugation, and normalized to equal protein concentrations as determined by BCA assay. A volume of 20 µL normalized mucus was applied in a uniform manner onto the membrane of air-liquid interface inserts, forming a continuous layer of mucus with a calculated thickness of approximately 606 µm. Influenza virus stocks were titrated in advance and 20 µL were applied to the apical side of the mucus layer at an infectious dose of 5 × 10^5^ FFU, while the lower compartment was filled with 500 µL culture medium (DMEM with 0.2% BSA). Inserts were incubated under humidified conditions at 37 °C with 5% CO₂ to allow virus penetration through the mucus barrier (Fig. 1C). At indicated time points, samples from the basolateral compartment were collected and viral penetration was quantified either by one-step RT-qPCR analysis of viral RNA or by infection of susceptible cells and subsequent immunofluorescent staining against IAV nucleoprotein (NP), followed by microscopic analysis. Viral penetration was expressed relative to the input virus concentration, enabling comparative analysis of penetration efficiency across different mucus samples or virus types, and normalized infection efficiency was calculated to quantify virus passage across the mucus barrier.

### Fluorescent staining and microscopic analysis

Fixed cells were incubated for 30 min at RT with permeabilization buffer consisting of PBS supplemented with 0.1% Triton X-100 and 0.2% BSA. Primary antibody against influenza virus nucleoprotein (NP) (Merck #MA8257) was diluted 1:500 in blocking buffer (PBS + 0.2% BSA) and added to the cells for either 1 h at RT or overnight at 4 °C. Afterwards, cells were washed three times with PBS. Secondary anti-mouse Alexa488 antibody (Invitrogen #A11032) was diluted 1:400 together with Alexa Fluor 647 conjugated phalloidin (Invitrogen #A22287) 1:400 in blocking buffer and added to the cells for 1 h at RT. Subsequently, cells were washed three times with PBS and stained with a 1 µg/mL Hoechst (Invitrogen #H1399) solution for 10 min. Cells were washed twice with PBS. Before mounting, cells were washed once with MilliQ water. Microscopic analysis was executed using an EVOS™ M500™ (Invitrogen™). We quantified experiments using the same imaging properties in one dataset. Images were acquired randomly, and each channel was saved as a separate TIF file. Infection was quantified using an automated image analysis pipeline in CellProfiler 4^34^.

### Quantification of influenza viral RNA by RT-qPCR

Viral RNA was extracted with the Quick-RNA Viral Kit (Zymo Research, R1035) according to the manufacturer’s instructions. Influenza A virus RNA was quantified by one-step RT-qPCR targeting the matrix gene (forward primer, 5′-GGCCCCCTCAAAGCCGA-3′; reverse primer, 5′-CGTCTACGYTGCAGTCC-3′)^48^. The one-step RT-qPCR protocol begins with reverse transcription, performed as a single cycle at 37 °C for 900 s (15 min). This is followed by RT inactivation and Hot-Start polymerase activation, also a single cycle, at 95 °C for 600 s (10 min). The reaction then proceeds through 50 cycles of PCR amplification, each consisting of three steps: denaturation at 95 °C for 10 s, annealing at 64 °C for 30 s, and elongation at 72 °C for 30 s. Finally, a melting curve analysis is performed, ramping the temperature up to 95 °C, to assess product specificity. A standard curve was generated from serial dilutions of a virus stock with a previously determined focus-forming titer, and results are reported as FFU equivalents (FFU eq). FFU eq represent viral RNA calibrated against the stock and are not a direct measure of infectious virus. Each run included no-template and no-reverse-transcriptase controls.

### Hemagglutination inhibition (HAI) assay and calculation of ${K}_{i}^{{HAI}}$

Hemagglutination inhibition assays were performed to evaluate the effect of mucus preparations on virus-mediated hemagglutination. Mucin matrices were adjusted to an equal O-glycan content and the corresponding protein concentration (µg/mL) was determined by BCA assay. For each compound, a two-fold serial dilution was prepared in PBS in round-bottom 96-well plates. An equal volume of influenza virus containing 4 hemagglutination units (HAU) was added to each well and the plates were incubated for 30 min at room temperature to allow binding. Subsequently, 50 µL of a 1.0% (v/v) suspension of chicken red blood cells in PBS was added to each well, and plates were incubated for 45 min at room temperature until the red blood cell controls formed a pellet at the bottom of the well. Wells were scored visually as inhibition (compact red “button”, no hemagglutination) or no inhibition (diffuse red layer, hemagglutination). For each mucus type, the HAI endpoint was defined as the highest dilution at which at least two of three replicate wells still showed complete inhibition. The inhibitory constant ${K}_{i}^{{HAI}}$ was then calculated from the initial protein concentration ${c}_{0}$ and the endpoint dilution factor ${D}_{{end}}$ using: ${K}_{i}^{{HAI}}=\frac{{c}_{0}}{{D}_{{end}}}$ and is reported in µg/mL. Lower ${K}_{i}^{{HAI}}$ values therefore indicate higher antiviral activity.

### Enzyme-linked lectin assay (ELLA)

Neuraminidase (NA) activity was quantified using an enzyme-linked lectin assay (ELLA). High-binding 96-well plates (ThermoScientific; 439454) were coated overnight at 4 °C with fetuin (25 µg/mL in coating buffer). Plates were washed with PBS containing 0.05% Tween-20 and blocked with blocking buffer (PBS, 1% BSA, 0.5% Tween-20). Virus stocks were two-fold serial diluted and transferred to the coated plates. Plates were incubated for 16–18 h at 37 °C in a humidified incubator with 5% CO₂ to allow NA cleavage of sialic acid end chains from fetuin. Plates were washed and incubated with horseradish peroxidase conjugated peanut agglutinin (PNA-HRP) for 2 h at room temperature in the dark, followed by development with o-phenylenediamine dihydrochloride (OPD). The reaction was stopped with 1 N sulfuric acid and absorbance was measured at 490 nm. For each virus, NA activity was recorded across the dilution series and background-corrected using no-virus control wells. To compare overall NA activity between virus types, the area under the curve (AUC) of OD490 versus virus dilution was calculated and used as a relative NA activity metric.

## Supplementary information

Rohde_et_al_Supplementary_Information_revised_final.

## Data Availability

All data supporting the findings of this study are available within the paper and its Supplementary Information. Data are available from the corresponding authors upon reasonable request.

## Declarations

### Journal association

Authors C.S. and D.C.L. are Associate Editors at ‚npj Viruses‘. C.S. and D.C.L. were not involved in the journal’s review of, or decisions related to, this manuscript.
