# Supplementary material for: Penetration of avian and human influenza A viruses through mucus gel networks in vitro
Source: Npj Viruses. 2026 Nov 11;4:57. doi: 10.1038/s44298-026-00257-7 (PMC13754920; doi:10.1038/s44298-026-00257-7)
Supplement: Supplementary file 1 [file 44298_2026_257_MOESM1_ESM.pdf]

Supplementary Information

Penetration of avian and human influenza A viruses through mucus gel networks in vitro

Maximilian Rohde[1], Fiona Riegel[1], Ege Duran[2], Cosmin Stefan Butnarasu[2#], Daniel Christian Lauster[2], Christian Sieben[1,3,4*]

[1] Helmholtz-Zentrum für Infektionsforschung GmbH, Braunschweig/Germany

[2] Institute of Pharmacy, Biopharmaceuticals, Freie Universität Berlin, Berlin/Germany

[3] Institut für Genetik, Technische Universität, Braunschweig/Germany

[4] BRICS - Braunschweig Integrated Centre of Systems Biology, Technische Universität, Braunschweig/Germany

[#] Current address: Department of Molecular Biotechnology and Health Science, University of Turin, Piazza Nizza 44/bis, 10126, Turin – Italy

[*] correspondence: christian.sieben@helmholtz-hzi.de

Keywords: Influenza A Virus, Respiratory mucus, virus-mucus interaction

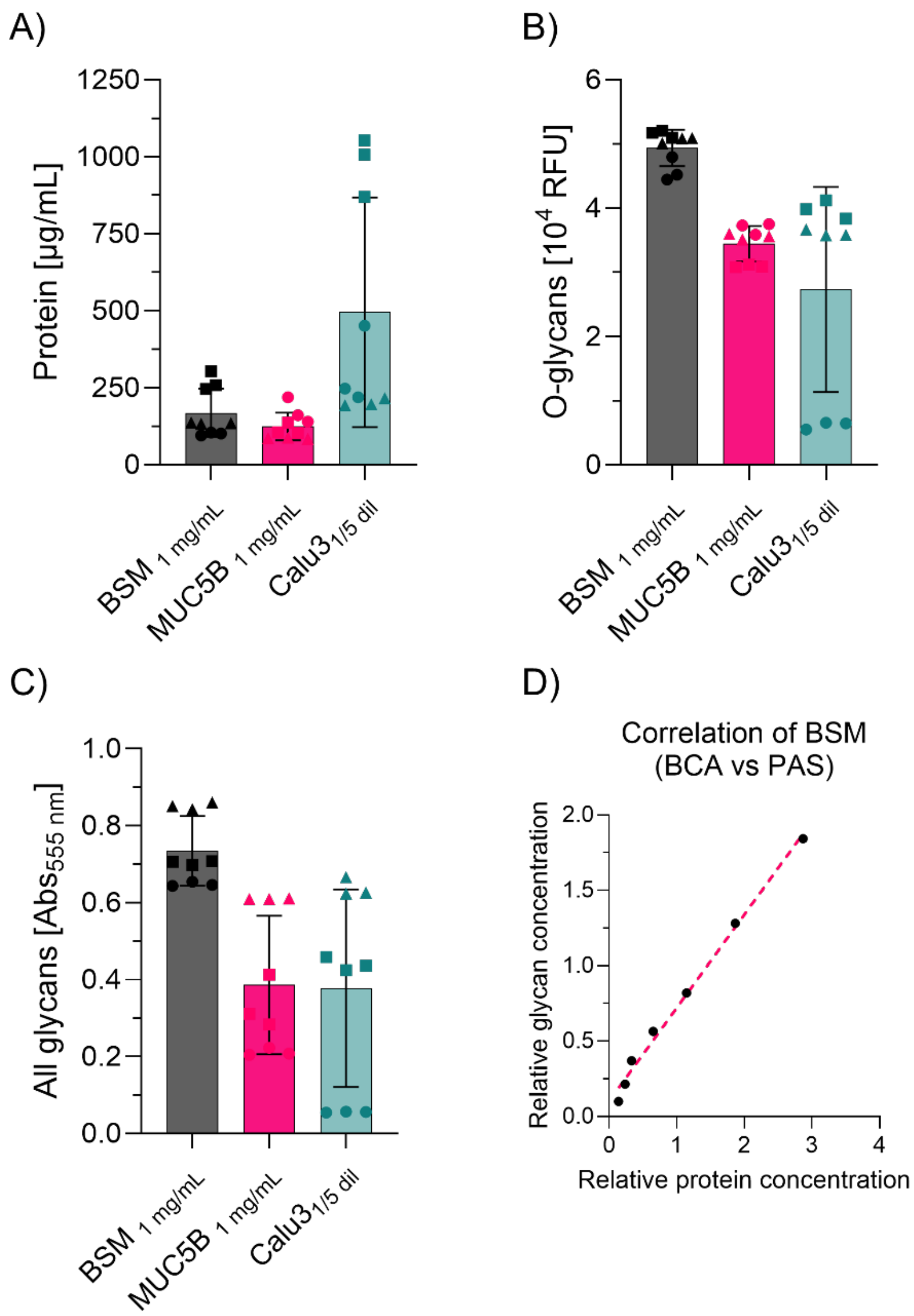

Figure S1: Representation of the values obtained from characterizing the mucus samples used. In this test 1 mg/ml BSM and purified MUC5B were compared to mucus harvested from Calu3 cells at a 1/5 dilution (approx. 550 µg/ml) **(A)**. Total protein content was measured using a commercially available BCA assay. **(B)** The O-glycan content is displayed in relative fluorescent units (RFU). **(C)** Glycan measurement was performed using a colorimetric PAS assay. **(D)** Visualized correlation of the protein and glycan content of a BSM dilution to demonstrate the linearity of these two factors. This phenomenon is also correlates between the protein content and o-glycan values.

```
NC_026433.1      MKAILVVLLYTFATANADTLCIGYHANNSTDTVDTVLEKNVTVTHSVNLLEDKHNGKLCK
H1N1pdm          MKAILVVLLYTFATANADTLCIGYHANNSTDTVDTVLEKNVTVTHSVNLLEDKHNGKLCK
                 ************************************************************

NC_026433.1      LRGVAPLHLGKCNIAGWILGNPECESLSTASSWSYIVETPSSDNGTCYPGDFIDYEELRE
H1N1pdm          LRGVAPLHLGKCNIAGWILGNPECESLSTASSWSYIVETPSSDNGTCYPGDFIDYEELRE
                 ************************************************************

NC_026433.1      QLSSVSSFERFEIFPKTSSWPNHDSNKGVTAACPHAGAKSFYKNLIWLVKKGNSYPKLSK
H1N1pdm          QLSSVSSFERFEIFPKTSSWPNHDSNKGVTAACPHAGAKSFYKNLIWLVKKGNSYPKLSK
                 ************************************************************

NC_026433.1      SYINDKGKEVLVLWGIHHPSTSADQQSLYQNADAYVFVGSSRYSKKFKPEIAIRPKVRXX
H1N1pdm          SYINDKGKEVLVLWGIHHPPTSADQQSLYQNADTYVFVGSSRYSKKFKPEIAIRPKVRXX
                 *******************.*************:**************************

NC_026433.1      EGRMNYYWTLVEPGDKITFEATGNLVVPRYAFAMERNAGSGIIISDTPVHDCNTTCQTPK
H1N1pdm          EGRMNYYWTLVEPGDKITFEATGNLVVPRYAFAMERNAGSGIIISDTPVHDCNTTCQTPK
                 ************************************************************

NC_026433.1      GAINTSLPFQNIHPITIGKCPKYVKSTKLRLATGLRNIPSIQSRGLFGAIAGFIEGGWTG
H1N1pdm          GAINTSLPFQNIHPITIGKCPKYVKSTKLRLATGLRNIPSIQSRGLFGAIAGFIEGGWTG
                 ************************************************************

NC_026433.1      MVDGWYGYHHQNEQGSGYAADLKSTQNAIDEITNKVNSVIEKMNTQFTAVGKEFNHLEKR
H1N1pdm          MVDGWYGYHHQNEQGSGYAADLKSTQNAIDEITNKVNSVIEKMNTQFTAVGKEFNHLEKR
                 ************************************************************

NC_026433.1      IENLNKKVDDGFLDIWTYNAELLVLLENERTLDYHDSNVKNLYEKVRSQLKNNAKEIGNG
H1N1pdm          IENLNKKVDDGFLDIWTYNAELLVLLENERTLDYHDSNVKNLYEKVRSQLKNNAKEIGNG
                 ************************************************************

NC_026433.1      CFEFYHKCDNTCMESVKNGTYDYPKYSEEAKLNREEIDGVKLESTRIYQILAIYSTVASS
H1N1pdm          CFEFYHKCDNTCMESVKNGTYDYPKYSEEAKLNREEIDGVKLESTRIYQILAIYSTVASS
                 ************************************************************

NC_026433.1      LVLVVSLGAISFWMCSNGSLQCRICI
H1N1pdm          LVLVVSLGAISFWMCSNGSLQCRICI
                 **************************
```

Figure S2: Amino acid alignment of the HA from pdmH1N1 used here with the reference genome sequence (EPI176620, NC_026433). Genomic RNA from pdmH1N1 virus used in this study was sequenced and the HA gene sequence was aligned against the HA gene from the reference genome EPI176620 (NC_026433).
